# Factors related to parents’ adherence to childhood immunization

**DOI:** 10.1186/s12889-022-13232-7

**Published:** 2022-04-25

**Authors:** Fatimah Hobani, Eman Alhalal

**Affiliations:** Nursing Collge, King Saud University, Riyadh, Saudi Arabia

**Keywords:** Childhood immunizations, Delayed immunizations, Missed immunizations, Saudi arabia, Health belief model

## Abstract

**Background:**

Immunizations protect children from deadly infectious diseases. Yet, there is still insufficient understanding of the factors associated with parents’ non-adherence to immunizations in contexts outside of Western countries. The aim of this study is twofold: (a) to investigate non-adherence to immunizations for children aged 6 months to 6 years in Saudi Arabia based on the number of immunizations missing or delayed by more than one month; and (b) to examine the underlying factors that predict the extent of non-adherence based on the Health Belief Model framework.

**Methods:**

A cross-sectional study was carried out in 22 randomly selected primary health care centers. Structured interviews were also conducted to collect data using the modified Health Belief Model questionnaire. Multiple regression analysis was used to assess the predictors of the extent of non-adherence.

**Results:**

Based on data from 220 participants, 51.8% of parents did not adhere with childhood immunizations. There was no significant relationship between parents’ sociodemographic characteristics and the extent of their hesitancy about children’s immunizations. The linear combination of perception of infectious disease severity, perception of their children’s susceptibility, perception of immunization benefits, perception of fewer barriers to obtaining immunizations, cues to action related to immunizations, and self-efficacy predicted the extent of non-adherence to immunizations (F (11.220) = 2.595, *p* < 0.001) and explained 12% of its variance. Yet, only perceived children’s susceptibility, perceived barriers, and self-efficacy independently predicted parents’ non-adherence.

**Conclusion:**

Saudi Arabia’s high proportion of non-adherence to childhood immunizations should be addressed. For instance, a health education program could be developed to increase parents’ awareness that their children are susceptible to health risks. Paying a special attention to existing barriers in accessing and receiving the immunizations is crucial. In addition, building parents’ self-efficacy, which is confident in making healthy decisions, such as keeping their children’s immunizations up to date, is important.

## Background

Most countries worldwide have implemented routine immunization programs as a public health approach [1], yet over 1.5 million children below the age of five die annually from vaccine-preventable diseases globally [2]. A low rate of vaccine coverage predisposes populations to preventable disease outbreaks [3]. Each vaccine is scheduled for a particular biological time frame when the child’s immune system can respond effectively and at the earliest to protect the child from the corresponding infectious disease [4]. Adherence to routine immunizations is crucial in minimizing the susceptibility to vaccine-preventable diseases and their outbreak [5, 6]. For example, during the pertussis outbreak in the United States of America (USA), the rate of infection was 3.2 times higher among unimmunized children compared with immunized children [7]. Adherence to vaccinations timelines is a very rigorous indicator of immunization status and population protection from infectious diseases [8]. Non-adherence includes not receiving vaccines at the age recommended by public health authorities [9] or refusal to receive some or all vaccines [10].

Parents’ fear of needles [11], vaccinations-related knowledge, employment status, educational level, economic status, family size [12], suspicion regarding vaccines effectiveness, forgetting appointments [13], lack of access to health care resources [14], and lack of self-efficacy [15] have been highlighted as factors related to adherence to immunizations. The internal and external motivating factors that encourage parents’ adherence, which are called cues to action [16], play an important role. For example, using media and technology to remind parents about vaccines can improve immunization adherence [17–19]. Confidence in making effective health decisions related to immunization uptake, which is called self-efficacy [20], might also be a factor that increases the likelihood of adherence to immunization [15, 21]. In a study of 18 European countries, some parents showed a significant lack of confidence regarding immunizations [22]. In general, most of the existing literature on parent/caregiver adherence to immunization originates from Western countries. Since attitudes and adherence to immunizations are shaped by broader sociocultural and attitudinal factors [23], as well as individuals’ psychosocial and economic and health contexts [24], these studies are not generalizable to countries outside of Western culture’s health-related belief systems, like Arab countries. Gaining a rich understanding of the factors that predict parent/caregiver adherence to routine childhood immunization is imperative for tailoring health interventions that tackle these factors, improve the likelihood of adherence to immunizations, and ultimately prevent the spread of infectious diseases.

Among Saudi children, 1.6% to 31.3% are unimmunized, 23.7% have received partial immunizations [25, 26], and 2.5% to 59.1% are behind schedule [27–29]. Studies in Saudi Arabia found that parents had limited information about the importance of immunizations [25], doubted their importance or effectiveness [28] or safety [25], and lacked access to vaccines for their children [29]. However, these studies did not use a theoretical framework and reliable scales to measure the factors associated with immunizations [25, 27–29]. Thus, there is a need to understand parents’ non-adherence to recommended children’s immunizations in the Saudi context with a focus on influential factors, using a theoretical framework.

The extent of individuals’ adherence to any preventive health behaviors might be influenced by their beliefs and attitudes toward that behavior [30]. The Health Belief Model (HBM) is a widely used theoretical framework for exploring why individuals do or do not engage in disease prevention actions [16, 20, 31]. The HBM consists of the following concepts: perceived severity, perceived children’s susceptibility (perceiving the possibility of acquiring health issues), perceived benefits (believing in the advantages of healthy behaviors), perceived barriers (perceiving physical and psychological barriers to health behaviors), cues to action (motivators that encourage healthy behaviors), and self-efficacy (beliefs about their abilities to pursue healthy behaviors) (Glanz et al., 2008).

**Fig. 1:**
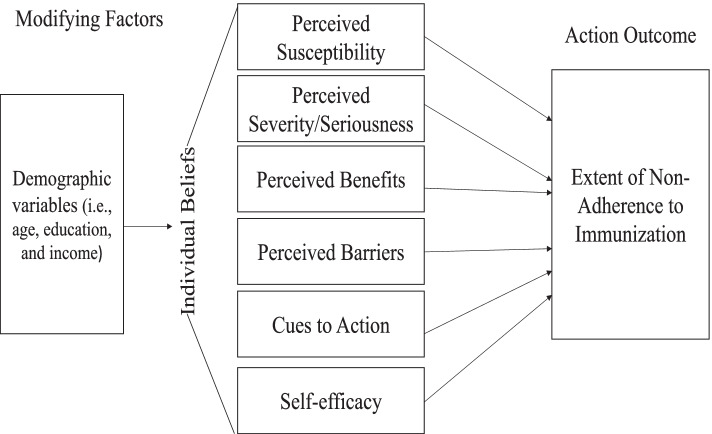
The hypothesize model based on the Health Belief Model

There are some modifying variables that may influence people’s decision to adopt healthy behaviors, such as demographics [32]. The HBM is suitable for examining parents’ adherence to routine childhood immunization because it considers cognitive factors and individuals’ beliefs related to their perceptions. It has been proven to be suitable for both population- and individual- based preventive interventions (Strecher & Rosenstock 1997). The HBM can guide the tailoring of interventions that address perceptions and tackle parents’ non-adherence to immunization. Based on the HBM [16], it was hypothesized that controlling for parents’ demographic characteristics (age, education, and income), parents’ perception of infectious disease severity, their children’s susceptibility, immunization benefits, barriers associated with vaccinations, cues to action related to immunizations, and self-efficacy predict the extent of their non-adherence to routine childhood immunizations (Fig. 1).

## Methods

### Aims

This study aims to 1) assess the prevalence of parents/caregiver non-adherence to immunizations for their children aged 6 months to 6 years based on the number of immunizations that are missing or delayed by more than one month; 2) examine the relationship between the extent of non-adherence to children’s immunizations and parent/caregiver demographic characteristics; and 3) explore the underlying factors that predict the extent of non-adherence to children’s immunizations using the HBM.

### Study design and setting

A cross-sectional study was conducted after obtaining approval from the Institutional Review Board of King Saud University and the Saudi Ministry of Health. In May and June 2020, 22 primary health care centers (PHCs) were randomly selected from Jizan area in Saudi Arabia. These centers are considered the first contact with the nation’s health care system and provide accessible and free curative, preventive, promotive, and rehabilitation services, including immunizations for all citizens [33].

### Participants and study procedure

The sample consisted of parents/caregivers with children aged 6 months to 6 years who were visiting the PHC for their child’s appointment. The sample size was calculated using G*power with an alpha value of 0.05, a power of 0.80, a medium effect size of 0.15, and a two-tailed linear regression test. The estimated minimum sample size was 208. As such, 220 participants were recruited. The response rate was 90.5%, based on the number of parents who agreed to participate out of those who were approached. The participants were recruited by in-person invitations during PHC visits, which contributed to the high response rate. After they were screened for eligibility, they gave their informed consent to take part in the study. All study data were collected using face-to-face structured interviews in private rooms at the PHCs. The researcher was asking the participants every question in a standardized order. Subsequently, copies of the children’s immunizations cards were obtained. The participants were assigned a unique ID number to ensure the anonymity and confidentiality of the data.

### Measures

For the parents’ demographic characteristics, the participants were asked to answer survey questions about their age, marital status, child relationship, family income, employment, and level of education. Also, the participants were asked to report their reasons of delayed and/or missed immunization. This single question is widely used in the literature [34–36]. The extent of non-adherence to childhood immunizations was assessed by checking the child’s immunizations record to determine the number of missing immunizations and the number of immunizations that were delayed by more than one month. This variable was constructed by summing the number of all missed and delayed immunizations.

A modified version of the HBM questionnaire [37] was used to measure the six concepts of HBM (predictors). For the purpose of this study, the HBM scale was adapted and translated into Arabic, following an integrated method for the scale’s adaptation according to population cultures and language [38]. As well, a pilot study was conducted with 30 parents to assess the reliability of the scale. Then, the scale was used in the main study with subscales’ reliability (internal consistency) based on a Cronbach’s alpha range of 0.813 to 0.612. The 30 responses obtained in the pilot study were not included in the final analysis.

The HBM scale has 35 items associated with the HBM concepts (perceived children’s susceptibility, perceived severity, perceived benefits, perceived barriers, cues to action, and self-efficacy). The perceived children’s susceptibility subscale has seven items that are responded to on a five-point Likert scale ranging from 1 “very unlikely” to 5 “very likely,” while the perceived severity/seriousness has eight items that are answered from 1 “very serious” to 5 “not serious.” The perceived benefits dimension comprises nine items based on a five-point Likert scale from 1 “very unlikely” to 5 “very likely.” The cues to action subscale with two items and the perceived barriers subscale with four items each have a five-point Likert scale ranging from 1 “strongly agree” to 5 “strongly disagree.” Finally, the perceived self-efficacy subscale has five items answered on a five-point Likert scale from 1 “very confident” to 5 “not very confident” [37]. Based on the scale guidelines, each subscale’s items were summed to produce the subscales’ scores (i.e., predictor variables). The internal consistency for each subscale was assessed, and the Cronbach’s alpha coefficients were 0.730, 0.813, 0.760, 0.803, 0.630, and 0.612 for perceived children’s susceptibility, perceived severity/seriousness, perceived benefits, and cues to action, perceived barriers, and self-efficacy, respectively. The final analysis included all items.

### Analysis

The data were analyzed using the Statistical Package for the Social Sciences software (SPSS) version 26. Descriptive statistics were produced for the participants’ demographic characteristics, all the variables, and the rate of non-adherence to children’s immunizations. All reported reasons of delayed and/or missed immunization as coded to calculate their frequency. The relationships between the demographic characteristics and parents/caregiver non-adherence were assessed using Pearson’s and Spearman’s rank-order correlation analysis as well as a two-tailed independent *t*-test. The hypothesized model was tested using multiple linear regression. The demographic characteristics were entered as control (confounding) variables in the regression model.

## Results

The parents’/caregivers’ demographic characteristics are shown in Table 1. The mean age of the fathers was 36.48 ± 6.152 years (range 25–55 years), while that of the mothers was 31.54 ± 6.072 years (range 18–46 years). In terms of education, half of the fathers (50%) and 71.4% of the mothers had completed university education. There were variations in family income (in Saudi Riyal (SAR)): lower than 5,000 (7.3%), 5,000 to 10,000 (32.3%), and 10,000 to 20,000 (50%; *n* = 110). In terms of occupational status, 93.6% of the fathers and only 39.5% (*n* = 87) of the mothers were employed. The mean number of children per family was 2.59 ± 1.457 (range: 1–7), and the mean age of the children was 33.6 ± 19.432 months. Their birth order ranged from 1^st^ to 7^th^ in the family, with a mean of 2.45 ± 1.45.

**Table 1:** Demographic characteristics of the survey participants (*N* = 220)

**Characteristic**	**Mean**	**SD**	**Range**
Mother age	31.54	6. 072	18–46
Father age	36.48	6.152	25–55
Child age (in month)	33.60	19.432	6–72
**Characteristic**	**Median (Mode)**	**Interquartile range**	**Range**
Number of the children in the family	2 (2)	3	1–7
Childbirth order	2 (1)	3	1–7
**Characteristic**		**Frequency (n)**	**Percentage (%)**
**Immunization status**			
Received all required vaccinations		198	90
Not received all required vaccinations (missing immunization)		22	10
Received up to date vaccinations		108	49.1
Received not up to date vaccinations (delayed)		112	50.9
**Accessibility to health care centers**			
Have not difficulty		202	91.8
Have difficulty		18	8.2
**Father level of education**			
Primary		4	1.8
Intermediate school		3	1.4
High school		69	31.4
Diploma		21	9.5
University		110	50
Postgraduate		13	5.9
**Mother level of education**			
Illiterate		1	0.5
Primary		3	1.4
Intermediate school		4	1.8
High school		32	14.5
Diploma		16	7.3
University		157	71.4
Postgraduate		7	3.2
**Family income (SR)**			
Less than 5000		16	7.3
Between 5000 to 10.000		71	32.3
From 10.000 to 20.000		110	50
More than 20.000		19	8.6
Social support		1	0.5
No income		3	1.4
**Father employment**			
Yes		206	93.6
No		12	5.5
**Mother employment**			
Yes		87	39.5
No		133	60.5

In terms of the extent of non-adherence, 51.8% of the children had at least one missed or delayed immunization. Table 2 presents the rate of delay for each vaccine, while Table 3 presents the reported reasons for delayed/missed vaccinations among non-adherent parents. It showed that the main reported reason for delayed immunization is forgetting the appointment (23.7%), followed by unavailability of the vaccines (18.4%). To elucidate the relationship between parents’ non-adherence and their demographic characteristics, correlation analyses, including Pearson’s correlation and Spearman’s rank-order correlation, were conducted (Table 4). Significant positive relationships were found between the extent of non-adherence and the higher number of children in the family, the child’s age, and the child’s birth order. A two-tailed independent-sample *t*-test showed no significant differences in the extent of non-adherence between employed and unemployed fathers (*t* (216) =  − 01.084, *P* = 0.895) or mothers (*t* (218) =  − 01.084, *P* = 0.280). The results of descriptive analyses for the independent variables (HBM concepts) and dependent variable (extent of non-adherence) are presented in Table 5.

**Table 2:** Delayed immunizations based on the age and type of vaccine (*N* = 220)

Type of vaccine	Frequency (n)	Percentage (%)
At birth BCG		
No delay	220	100
2 Months (1^st^ DTP + 1^st^ IPV + 1^st^ Hib + 1^st^ hepatitis B + 1^st^ PCV + 1^st^ Rota)		
No delay	210	95.5
One month delay	3	1.4
Two months delay	2	0.9
Three months delay	1	0.5
Four months and more delay	4	1.8
4 Months (2^nd^ DTP + 2^nd^ IPV + 2^nd^ Hib + 2^nd^ hepatitis B + 2^nd^ PCV + 2^nd^ Rota)		
No delay	190	86.4
One month delay	19	8.6
Two months delay	7	3.2
Three months delay	1	0.5
Four months and more delay	3	1.4
6 Months (3^rd^ DTP + 1^st^ OPV + 3^rd^ IPV + 3^rd^ Hib + 3^rd^ hepatitis B + 3^rd^ PCV)		
No delay	183	83.2
One month delay	24	10.9
Two months delay	6	2.7
Three months delay	1	0.5
Four months and more delay	6	2.7
9 Months (Measles + MCV4)		
No delay	189	85.9
One month delay	18	8.2
Two months delay	4	1.8
Three months delay	7	3.2
Four months and more delay	2	0.9
12 Months (1^st^ MMR + 4^th^ PCV + 2^nd^ MCV4 + 2^nd^ OPV)		
No delay	174	74.5
One month delay	22	10
Two months delay	14	6.4
Three months delay	7	3.2
Four months and more delay	13	5.9
18 Months (2^nd^ MMR + 3^rd^ OPV + 4^th^ DTP + 1^st^ + 4^th^ Hib + hepatitis B + Hepatitis A + 1^st^ Varicella)		
No delay	169	76.8
One month delay	18	8.2
Two months delay	16	7.3
Three months delay	6	2.7
Four months and more delay	11	5
24 Months (2^nd^ Hepatitis A)		
No delay	170	77.3
One month delay	10	4.5
Two months delay	5	2.3
Three months delay	7	3.2
Four months and more delay	28	12.7

*BCG* Bacille Calmette-Guerin, vaccine/ tuberculosis, *OPV* Oral Polio Vaccine, *IPV* Inactivated Polio Vaccine, *DTP* Diphtheria, Tetanus, Pertussis, *Hib* Haemophilus Influenzae type b, *PCV* Pneumococcal Vaccine, (Quadrivalent) *MCV4* Meningococcal Conjugate Vaccine, *MMR* Measles, Mumps, Rubella

**Table 3:** Reasons for delayed immunization among non-adherent group (*N* = 114)

Reported Reasons for Delayed Immunization	Frequency (n)	Percentage (%)
Forget child appointment	27	23.7%
Unavailability of the Vaccines	21	18.4%
Child Sick	16	14%
Family Circumstances	8	7.0%
COVID 19	8	7.0%
Accessibility	7	6.1%
Neglect	6	5.3%
Hesitate	3	2.6%
Mothers Sick	2	1.8%
No Appointment	2	1.8%
Others (No Reason Given)	14	12.3%

**Table 4:** The relationship between the background characteristics and the extent of non-adherence (*N* = 220)

**Variables**	**Correlation**	***P value***
No. of children	0.192**	0.004
Child Age	0.172*	0.011
Father Age	0.050	0.464
Mother Age	0.054	0.428
Family Income (SAR)	0.035	0.605
Childbirth Order	0.179**	0.008
Fathers Education	0.086	0.201
Mothers Education	0.010	0.884
Marital Status	0.106	0.116
Fathers Job	0.010	0.886
Mothers Job	0.057	0.397

**p < 0.01, *p < 0.05 (2-tailed)

**Table 5:** Descriptive statistics of the study variables (*N* = 220)

HBM subscales (score)	Mean	SD	Skewness		kurtosis	
			**Statistic**	**Std error**	**Statistic**	**Std error**
Perceived susceptibility (7 to 35)	17.14	4.165	-0.010	0.164	0.066	0.327
Perceived severity (8 to 40)	20.28	6.575	0.586	0.164	0. 021	0.327
Perceived benefits (9 to 45)	33.68	5.370	-0.357	0.164	0.276	0.327
Cues to action (2 to 10)	4.35	2.304	0.883	0.164	-0.131	0.327
Perceived barriers (4 to 20)	15.61	3.287	-1.046	0.164	1.667	0.327
Self-efficacy (5 to 25)	10.09	3.302	0.596	0.164	0.768	0.327
Extent of non-adherence	3.409	4.717	1.725	0.164	3.270	0.327

## Testing the study hypothesis

Guided by HBM, multiple linear regression was used to test the hypothesized model in which caregivers’/parents’ perceptions of infectious disease severity, their children’s susceptibility, immunizations benefits, immunizations-related barriers, cues to action related to immunizations, and self-efficacy predicted the extent of adherence to immunizations, controlling for parents’ age, income and education. The results demonstrated that the overall model was significant (*F* (11, 220) = 2.95, *p* < 0.001) and 12% of the variance in the extent of non-adherence was explained by the linear combination of the HBM’s six concepts, with controlling for parental demographic factors. Three out of the six predictors were considered to be individually significant in predicting the extent of non-adherence. These predictors were perceived children’s susceptibility ($\upbeta$ = − 0.1512, *P* = 0.025), perceived barriers ($\upbeta$ = 0.216, *P* = 0.002), and self-efficacy ($\upbeta$ = − 0.158, *P* = 0.018). Perceived severity, perceived benefits, and cues to action were not significant, unique predictors of the extent of non-adherence. The regression analysis results are presented in Table 6.

**Table 6:** Linear regression analysis (*N* = 220)

HBM Subscales	Unstandardized coefficients		Standardized coefficients		95% Confidence Interval (CI)
	**B**	**SE**	**β**	**P**	
Perceived Susceptibility	-0.151	0.067	-0.152	0.025	-0.282 – -0.019
Perceived Severity	-0.043	0.043	-0.069	0.319	-0.128 – 0.042
Perceived Benefits	-0.033	0.050	-0.044	0.506	-0.132 – 0.066
Cues to Action	0.187	0.120	0.105	0.120	-0.049 – 0.423
Perceived Barriers	0.267	0.084	0.216	0.002	0.433 – 0.102
Self-efficacy	0.198	0.083	0.158	0.018	0.034 – 0.362

## Discussion

Guided by the HBM, this study is one of the few to assess parents/caregiver non-adherence to their children’s immunizations in an Arab context such as Saudi Arabia. We found support for the hypothesis that was derived from the HBM. First, 51.8% of parents did not adhere to the Saudi recommended children immunization schedule, by having delayed or missed vaccinations. This finding is inconsistent with the high coverage in Saudi Arabia (96% to 98%) [39]. In other words, the high immunization coverage does not reflect poor adherence to the immunization schedule, as the children appear to be receiving all of their immunizations, but not on timely way. With high coverage or uptake but poor adherence to the immunization schedule (e.g., delayed immunization), there are negative consequences becauseg children are more likely to be exposed to harmful pathogens. Thus, the coverage rate is inadequate to understand population immunity. Our study sheds light on non-adherence as an existing issue even with Saudi Arabia’s high coverage rate and the availability of a free routine immunizations program that is offered to all citizens. This means the coverage rate might overestimate the population protection and is not a good indicator for the real dynamics of children’s immunizations [40], as 50.9% of parents in our study had delays in their children’s immunizations. This study highlights the fact that a high proportion of children aged 6 months to 6 years are not immunized based on age-appropriate vaccines. Following the immunization schedule timeline ensures the prevention of diseases in the community [40]. Thus, there is a crucial need to focus public health efforts on addressing timeline adherence.

Our findings are consistent with those of studies in other parts of the world with different socioeconomic contexts. For example, in the USA, among children under five years, 74% of children received at least one dose late [41]; in Australia, 20% of children have delayed immunizations [42]; and in Belgium, 32%–95% of children have delayed immunizations [43]. The previous studies that were conducted in Saudi Arabia revealed that the rate of non-adherence was 22.4% to 59.0% for late immunizations [27, 29, 44], which is consistent with our findings.

The most common delayed vaccines are for Measles Mumps Rubella (MMR) and the 2^nd^ meningococcal conjugate vaccine (MCV4), 2^nd^ Oral Polio Vaccine (OPV), and 4^th^ pneumococcal conjugate (PCV) vaccine given at 12 months. These vaccines are also reported as the most delayed in another study in Saudi Arabia [45]. The main reason of this delay is not clear. The most commonly reported reason for delayed immunizations in this study was forgetting the child’s appointment. In other parts of the world, forgetting an appointment was not the most reported reason for delayed immunizations [46, 47]. Therefore, an intervention that includes reminding and calling systems might be needed in the Saudi context to increase timeline adherence to routine childhood immunizations [48].

A positive association was found between the extent of non-adherence and having greater number of children in the family. It means an attention should be paid to children immunization with larger families as this relationship was also found in the literature [49, 50]. Parents with more children might have a lot of responsibilities which limit their ability to allocate time and subsequently district their adherence. Moreover, higher child’s birth order increases non-adherence which is also consistent with the existing evidence [51]. Parents with more children tend not feel the urgency due to the fact that their older children never get any vaccine-preventable disease [51]. Also, an association was found between non-adherence and child age, as the non-adherence increases when children get older [52]. It might be explained by previous studies which highlighted that as children get older, parents’ non-adherence to immunization increases due to needle fears [11] and previous experience with side effects (i.e., fever and pain) [6]. However, further studies are needed to explain these relationships.

This study found that the extent of non-adherence was not related to parents’ demographic characteristics like age, income, education, and employment status. This finding contradicts the common assumption that non-adherence is due to parents’ poor socioeconomic status. Comparisons with existing studies are challenging due to inconsistencies across contexts. Some studied found no significant associations between parents’ level of education, age [13], and income [47] and their children’s immunizations completion, whereas other studies found significant associations between parental education [53–55], age [9], and income [46, 53, 55, 56] and immunizations adherence [54, 56]. These inconsistencies might be a result of the differences in sociocultural contexts between countries. The health care system in Saudi Arabia provides free public health services, and its primary health care system is built to be accessible to all citizens by providing highly efficient curative and preventive services, such as immunizations [33]. Thus, all citizens access the primary health care regardless of their socioeconomic status, as confirmed in a study that found income and education did not significantly predict primary care utilization in Saudi Arabia [57]. The current sample characteristics are almost similar to the general population; for example, in the sample, 39.5% of mothers were not employed, while in the general population 33% of women are employed. However, we can’t generalize the findings that socioeconomic status is not related to vaccine adherence given the current limited sample size.

The hypothesized model based on the HBM significantly predicted the extent of non-adherence to childhood immunizations among Saudi parents. This means that the HBM is suitable for understanding non-adherence to immunizations, and this is in alignment with previous studies that found it can predict children’s immunizations in the USA [58] and China [59]. The HBM is beneficial in predicting engagement or lack of engagement in proactive preventive health behaviors based on its six concepts (perceived children’s susceptibility, perceived severity, perceived benefits, perceived barriers, cues to action, and self-efficacy). Thus, the results of this study contribute to understanding the importance of tailoring programs that consider public perceptions of infectious disease severity, children’s susceptibility, immunizations benefits, and barriers related to immunizations, as well as cues to action related to immunizations and parents’ self-efficacy, in order to address parents’ non-adherence to immunizations. However, looking to these factors individually, we found that perceived children’s susceptibility, perceived barriers, and self-efficacy were significant predictors of parents’ non-adherence.

The findings that perceived barrier was a significant predictor, is in alignment with the reported reasons by participants (inaccessibility, unavailability of some vaccines and appointment availability). Barriers of accessing the health care system includes the inability to reach and obtain appropriate health care resources, unacceptability, and inadequacy of the service [60]. The top reported reason for delayed immunization was forgetting the appointment. Thus, the use of reminder/recall services is effective in improving immunizations rates [61]. The second reported reason was unavailability of some vaccines during specific times and this could be considered as a barrier as some centers’ demands might exceed supply. Although health services such as immunization are offered freely to citizens in Saudi Arabia, 6.1% of parents reported inaccessibility as a reason. Reporting inaccessibility could be related to transportation and the distance of the centers. In crowded neighborhoods or PHCs, it may be difficult to schedule an appointment, yet only 1.8% reported lack of appointment availability. Also, although only 7% of non-adherent parents reported avoiding visiting clinics due to COVID-19, the pandemic itself might be a barrier in adhering with routine childhood immunization [62]. The finding that controlling for other factors, perceived barrier is still a significant factor in parents’ non-adherence is an important finding. However, further studies are needed to identify parents’ barriers to adhering to childhood immunization. These studies can be used to inform public health policies about strategies to reduce barriers.

The perceptions of parents regarding their children’s susceptibility to diseases and possibility of suffering from a specific health issue predicted the parents’ non-adherence to routine immunizations. This finding accords with that of a study that reported susceptibility significantly predicted parents’ intention to have their children immunized against influenza [59]. The fact that the perception of children’s susceptibility predicts parents’ non-adherence demonstrates the importance of parents’ understanding the risk of their children contracting vaccine-preventable diseases if they are not immunized. Health education programs, mass media, and campaigns can be developed to focus on increasing parents’ awareness of such risks.

Self-efficacy, which concerns parents’ confidence in making decisions about their child’s immunization, was also a significant predictor of the extent of non-adherence. This finding is consistent with a study in East Asia, which found that mothers’ influence on decision-making and self-efficacy increased the probability of vaccinating their children [15]. However, they had good perceptions guiding their decisions to adapt healthy behaviors. In our study, the self-efficacy of parents was predictive of non-adherence, suggesting that health care providers and national strategies should target parents’ confidence to uptake routine childhood immunizations. In Saudi Arabia, studies have confirmed parents’ confidence in children’s immunizations [26]. In contrast, a study in South Korea revealed that issues related to self-efficacy were not significant predictors of the intention to immunize children [63]. Self-efficacy, where there is confidence in health decision to improve health and quality of life, might increase parents’ perseverance to overcome difficulties and comply with recommendations for childhood immunizations. However, in our study, when self-efficacy increased, non-adherence increased as well. This might be explained by the fact that parents who were confident about immunizations believed it would be acceptable to postpone them and catch up later. Self-efficacy might also be associated with receiving false information, leading to non-adherence to immunization. Further studies are needed to explore why parents’ self-efficacy is related to non-adherence to immunizations.

This study has a few limitations. For instance, the use of a convenience sample limits the generalizability of the findings to the entire population. The sample was collected from only one city in Saudi Arabia; as such, it is not representative of the Saudi population. There might be socioeconomic differences between the sample from Jizan and other cities in Saudi Arabia. Thus, future research should consider recruiting participants from different regions in the country through random sampling. Since data was collected during COVID-19, a context without the pandemic might yield different results concerning adherence, and this could limit the generalizability of the current findings. Finally, this study used a cross-sectional study design, which does not permit individuals to draw conclusions about the causality of the emergent predictive relationship. Although the study translated and tested the HBM scale by recruiting a pilot sample of 30 parents/caregivers who visited PHCs, the validity and reliability of the scale are limited because this is the first study to apply it in an Arab or Saudi context.

## Conclusion

The extent of parents’ non-adherence to immunizations in Saudi Arabia was found to be relatively high, indicating a need for special attention to missing or delayed vaccines and immunization timelines. Parents’ perception of children’s susceptibility, perceived barriers, and self-efficacy were significant factors influencing adherence to childhood immunizations. Therefore, there is a need to develop national strategies and tailor health programs to address these factors for the purposes of preventing infectious diseases and protecting children’s health. The findings can be generalized to a context that is similar to Saudi Arabia.

## Data Availability

The study datasets are available from the corresponding author on reasonable request.

## Abbreviations

PHC: Primary Health Care
HBM: Health Belief Model
MMR: Measles, Mumps, and Rubella
PCV: Pneumococcal Conjugate Vaccine
MCV: Meningococcal Conjugate Vaccine
OPV: Oral Polio Vaccine
